# Target genes of myostatin loss-of-function in muscles of late bovine fetuses

**DOI:** 10.1186/1471-2164-8-63

**Published:** 2007-03-01

**Authors:** Isabelle Cassar-Malek, Florent Passelaigue, Carine Bernard, Jean Léger, Jean-François Hocquette

**Affiliations:** 1 Equipe Croissance et Métabolisme du Muscle, Unité de Recherche sur les Herbivores, UR1213, INRA Theix, 63122 Saint-Genès-Champanelle, France; 2 PT transcriptome, Ouest Génopole, Institut du Thorax, Faculté de Médecine 1, rue Gaston Veil, 44035 Nantes cedex, France Faculté de Médecine 1, rue Gaston Veil, 44035 Nantes cedex, France

## Abstract

**Background:**

Myostatin, a muscle-specific member of the Transforming Growth Factor beta family, negatively regulates muscle development. Double-muscled (DM) cattle have a loss-of-function mutation in their myostatin gene responsible for the hypermuscular phenotype. Thus, these animals are a good model for understanding the mechanisms underpinning muscular hypertrophy. In order to identify individual genes or networks that may be myostatin targets, we looked for genes that were differentially expressed between DM and normal (NM) animals (n = 3 per group) in the *semitendinosus *muscle (hypertrophied in DM animals) at 260 days of fetal development (when the biochemical differentiation of muscle is intensive). A heterologous microarray (human and murine oligonucleotide sequences) of around 6,000 genes expressed in muscle was used.

**Results:**

Many genes were found to be differentially expressed according to genetic type (some with a more than 5-fold change), and according to the presence of one or two functional myostatin allele(s). They belonged to various functional categories. The genes down-regulated in DM fetuses were mainly those encoding extracellular matrix proteins, slow contractile proteins and ribosomal proteins. The genes up-regulated in DM fetuses were mainly involved in the regulation of transcription, cell cycle/apoptosis, translation or DNA metabolism. These data highlight features indicating that DM muscle is shifted towards a more glycolytic metabolism, and has an altered extracellular matrix composition (e.g. down-regulation of *COL1A1 *and *COL1A2*, and up-regulation of *COL4A2*) and decreased adipocyte differentiation (down-regulation of *C1QTNF3*). The altered gene expression in the three major muscle compartments (fibers, connective tissue and intramuscular adipose tissue) is consistent with the well-known characteristics of DM cattle. In addition, novel potential targets of the myostatin gene were identified (MB, PLN, troponins, *ZFHX1B*).

**Conclusion:**

Thus, the myostatin loss-of-function mutation affected several physiological processes involved in the development and determination of the functional characteristics of muscle tissue.

## Background

Studies during the past decade have shown that the product of the gene *myostatin *(*GDF8*) (a muscle-specific TGFβ family member) is an inhibitor of muscle development and of the maintenance of muscle mass. Mutations in *myostatin *[1,2] result in double-muscling (DM) in both cattle and rodents. In cattle, several disruptive *myostatin *mutations have been identified in different breeds [3,4]. These mutations truncate the protein product resulting in functional inactivation. For example, in the Belgian Blue breed, an 11-bp deletion [nt821 (del11)] has occurred in the third exon in a region encoding the bioactive domain. Similarly, the Q204X mutation (a C to T transition), which results in a premature stop codon in the N-terminal LAP (Latency Associated Peptide) domain, is frequently found in the Charolais breed or in the INRA95 genotype [4].

*Myostatin-*null mice exhibit enlarged skeletal muscles relative to wild-type littermates because the numbers (hyperplasia) and area (hypertrophy) of muscle fibers are increased [1]. In cattle breeds, double-muscling is primarily due to hyperplasia [5] as early as the fetal period [6,7]. *Myostatin *expression is regulated throughout gestation [2,8]. It was found to be located in the most recently differentiating cells throughout bovine fetal development [9]. Myostatin may negatively regulate the number of fast-glycolytic (IIX) fibers, which is therefore increased in DM muscles at the expense of oxidative fibers [8]. Furthermore, the properties of DM muscles differ from those of normal ones (NM) owing to lower collagen and intramuscular fat contents [10]. In adult muscle, *myostatin *is specifically expressed in satellite cells and behaves as an important regulator of satellite cell activation and renewal, thereby controlling muscle mass (reviewed in [11,12]).

Examination of the molecular action of myostatin has revealed that it inhibits the proliferation of myogenic cells through the control of cell cycle progression [13]. This provides an explanation for the higher proliferation rates of DM fetal myoblasts than controls *in vitro *[8,14]. Myostatin also protects myoblasts from apoptosis and delays their terminal differentiation [15,16]. Functional myostatin binds to the activin type IIB transmembrane receptor, which then recruits and activates the ALK type I co-receptor by phosphorylation. This results in recruitment of the SMAD signaling pathway [17]. An alternative pathway involving p38 MAPK signaling has also been proposed to account for growth inhibition [18]. The inhibition of myogenesis is mediated partially through a decreased expression of Myogenic Regulatory Factors (reviewed in [12]). Myogenin and p21CKI have been identified as the major physiological targets of endogenous myostatin in murine cells [19]. Proteomics has also revealed novel differentially-expressed proteins associated with double-muscling in bull calves [20], suggesting the existence of unidentified myostatin targets.

In order to identify differences in gene expression, and hence novel genes or networks that may be myostatin targets liable to be involved in muscle differentiation, we examined the transcriptional profiling of the *semitendinosus *(ST) muscle of DM fetuses *vs *Non-Double-Muscled (NM) at 260 days of gestation.

## Results and Discussion

### Microarray experiments

**Figure 1:**
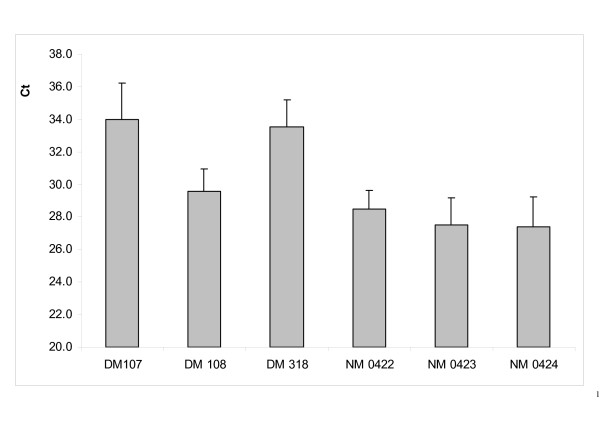
Myostatin expression in the ST muscle of experimental fetuses. Myostatin expression was assessed by quantitative RT-PCR using the Sybergreen method. Values are means ± S.E. for n = 6. The highest crossing point (Ct) corresponds to the lowest expression level. DM: double-muscled fetus; NM: non-double-muscled fetus. DM108 is heterozygous for the Q204X mutation.

We performed microarray analyses of the ST muscle of DM of the INRA95 genotype and NM fetuses (n = 3 per group) using heterologous oligonucleotide chips (Myochips, human and murine sequences) dedicated to muscle and cardiac gene expression. The ST muscle was chosen since it is highly hypertrophied in DM. The rationale for studying gene expression in late fetuses was that expression of many genes is affected during the last third of gestation [21], a key period in the fiber differentiation/specialization process in cattle, which are mature at birth with regard to muscle physiology [22]. The arrays have the advantage of high quality and specificity together with a large number of genes. Some 75% of their oligonucleotide sequences are human, which was another advantage for the study since the comparative coverage of the bovine and human genomes is about 91% [23]. Myostatin expression in ST muscle was monitored by quantitative RT-PCR (Figure 1) and was found to be lower in DM than NM muscle. However, it was more elevated in one of the DM foetuses, which was found to be heterozygous for the Q204X mutation.

Hybridization of bovine targets on to the Myochips enabled us to recover 75–84% of valid expression values. Examination of microarray data from DM and NM fetuses allowed us to identify major differences in gene expression amongst individuals and genotypes.

**Figure 2:**
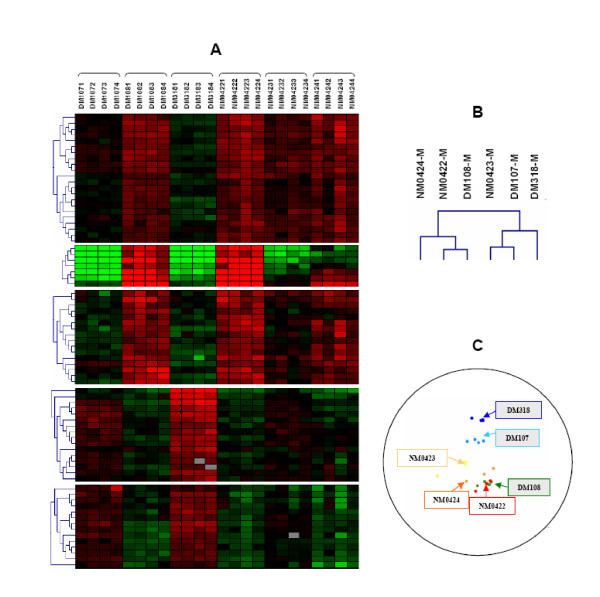
Hierarchical clustering and Principal Components Analysis of expression data reveal the influence of heterozygosity for the Q204X mutation and the variability of gene expression among individuals. Four individual arrays were used per animal. Clustering (A, B) and PCA (C) were performed using Genesis and GenANOVA respectively. (A): Hierarchical clustering of genes – each column represents an individual array; (B): Hierarchical clustering of animals' mean (-M) expression data; (C): PCA of animals, each projection is representative of an individual array; DM: double-muscled fetus; NM: non-double-muscled fetus. DM108 is  heterozygous for the Q204X mutation.

Hierarchical clustering of the data allowed the genotype groups to be clearly discriminated (DM/NM, Figure 2). However, it revealed a transcriptional profile for the heterozygote DM108 fetus more closely related to that of the NM animals (Figure 2A, B). Principal component analysis confirmed this finding (Figure 2C). Thus, muscle gene expression appeared to be altered in fetuses harbouring the Q204X mutation. However, this influence differed according to whether one or two impaired myostatin allele(s) were present, illustrating the autosomal recessive character of *myostatin *in cattle [24], as already shown for muscle protein expression [20].

Expression data were filtered for 20% missing values and processed by ANOVA. Eight clusters were selected. They included 189 genes the expression of which varied according to the presence of one or two functional myostatin allele(s) as shown by the hierarchical clustering. Only 142 of them had a GO annotation. Some of these could be potential candidate targets of functional myostatin. Twelve were also found to be differentially expressed postnatally in the ST muscle of DM vs NM cows (our unpublished data). Using FatiGO+, an evolution of FatiGO, we searched for GO functional annotation and the KEGG pathway. Some genes were annotated for carbohydrate metabolism (e.g. *Foxc2*, *SDS*, *APM1*), for lipid, fatty acid and steroid metabolism (e.g. *PLIN*, *APM1*), or for protein metabolism and modification (e.g. *Mrpl36*, *CTBP1*, *PAK1*, *SMAP1*). Interestingly, these putative myostatin targets were predicted to belong to 51 different KEGG pathways (Table 1) such as focal adhesion, axon guidance, calcium signaling pathway, cell cycle, or lastly Wnt signaling, which was recently shown to be altered in *myostatin *knocknull-out mice [25].

### Differentially expressed genes according to myostatin loss-of-function

Using SAM, a differential analysis of the hybridization data was carried out between the two groups of three fetuses (dataset 1, Table 2). It was also performed between two groups of two extreme fetuses (dataset 2 excluding the DM108 heterozygote and an NM0423 animal, the myostatin expression and gene profiling of which were intermediate) to maximize the difference between the two genotypes (Table 2). The latter analysis was chosen in order to identify true differentially expressed genes with confidence. Analyses allowed a false discovery rate (FDR) that accepts that 5‰ of the genes declared differentially expressed will be false positives. In both analyses, a substantial number of genes were differentially expressed and this number varied according to the fold change value (FC). More than 93% of the genes identified from dataset 1 were declared to be differentially expressed from dataset 2 (Table 2). Taking a FC ≥ 2, the same genes were identified in both datasets. However, dataset 2 allowed an additional 53 down- and 86 up-regulated genes to be identified with a FC ≥ 1.4 (Table 2). The rationale for taking differential expression with a FC ≥ 1.4 and FDR < 5‰ into account was to ensure that greater numbers of differential genes were retained with high confidence, using 4 technical replications per animal.

Examples of genes declared differential by SAM from dataset 2 are presented in Table 3 (up-regulated genes) and Table 4 (down-regulated genes). ANOVA confirmed that the expression of 93% of these genes was statistically significant (F>11, *p *< 0.005). The genes with FC ≥ 2 had *p-values *< 5‰ by ANOVA, except one (*2410044K02Rik*, *p-value *< 0.02). Some differential expressions were confirmed by real-time RT-PCR (Table 5). A search for homology between the oligonucleotides representing differential genes with a FC = 1.4 and the bovine genome was carried out using BLASTN and BLASTX searches. More than 80% of the oligonucleotides were found to have a homology greater than 70% with bovine sequences (data presented partially in Tables 3 and 4). These data confirmed the appropriateness of the oligochips even though they were made of human and murine oligonucleotide sequences.

### Function of differential genes in DM and biological relevance

The data were further explored using Gene Ontology (GO) information (Biological Process and Molecular Function terms). This predicted that 95 of the down-regulated genes with GO annotations encoded mainly ribosomal proteins, ECM/cell interaction proteins or sarcomeric slow contractile proteins. Conversely, the genes up-regulated in DM muscle were annoted for regulation of the cell cycle, transcription and DNA metabolism. Only 136 of the 242 up-regulated genes had a GO annotation at the Biological Process level. Of these, 30 were annotated for "regulation of transcription", of which 24 were annotated with the GO term "Transcription Factor". They included regulators of muscle-specific gene expression such as *MEF2A, ID1 *or *ZFX1B (ZEB2/SIP1) *and also *MyoD1 *for which the FC (1.35) was just under our FC threshold (1.4).

Some down-regulated genes were found to belong to KEGG pathways (Table 6), e.g. the ribosome, oxidative phosphorylation and ATP synthesis, calcium signaling pathway and extracellular matrix (ECM)/receptor interaction. Up-regulated genes were mainly involved in the insulin pathway, cytokine/receptor interaction, Wnt signaling, cell cycle, apoptosis and axon guidance (Table 6). Altogether, these results indicated that myostatin loss-of-function was associated with the alteration of many biological pathways in DM muscle.

#### Genes involved in protein metabolism

One notable finding of our study was that an important subset of the differential genes was involved in protein metabolism or encoded ribosomal proteins. Interestingly, some authors have specifically looked for genes that are differentially regulated in early DM embryos compared to normal ones [26]. As in the present study, they identified differential expression of ribosomal proteins, suggesting that NM and DM animals may differ in protein degradation and synthesis.

#### Genes involved in contractile and metabolic function

The functional categories of the genes down-regulated in DM illustrate the so-called phenotypic muscle characteristics of these animals. First, the findings highlighted a marked down-regulation of genes encoding slow contractile proteins in fibers (e.g. *cardiac *and *slow troponin C *and *T *isoforms, *MYH7 *and *MYL2*, *TPM3*; Table 4), slow twitch proteins (e.g. *PLN*, and *SERCA2*; Table 4) and *MB *(*myoglobin*), the differential expression of some of these genes being confirmed by real-time RT-PCR (Table 5). The Bibliosphere Pathway Edition web tool of the Genomatix Suite predicted that, in rodents or humans, some of the down-regulated genes (*SERCA2, MyH7, S100A4, VIM, FN1, COL1A2*) had an NFKB1 site in their promoter. Interestingly, the *NFKB1 *gene was found to be up-regulated in DM (FC = 1.33, FDR < 5‰, p value < 5‰), suggesting that NFKB1 expression could contribute to negative regulation of their expression as already shown for collagen *COL1A2 *[27]. Other genes involved in contraction (*MYH7, MB, TNN C, desmin*) were also predicted to be targets of TEF-1 at MEF2 elements during fast-to-slow muscle conversion, as reported for humans [28].

Conversely, the study also showed up-regulation of *slc16a10 *(Table 3), which encodes a transporter catalyzing the transport of many monocarboxylates including lactate and pyruvate [29], and of *LDH-A*, which encodes lactate dehydrogenase, although with an FC below our threshold (1.37). Moreover, it confirmed that the expression of *MyBP-H*, which encodes a component of the thick filaments in fast skeletal muscles, was up-regulated (Table 3) in the ST muscle of DM fetuses, as shown postnatally by proteomic approaches [20]. All in all, these data indicated that DM muscles were shifted towards a more glycolytic fast metabolism. A similar observation was reported in *myostatin-*null mice [25,30]. Moreover, it has been demonstrated that the muscles of DM cattle contain a higher proportion of fast glycolytic IIX fibers and a lower proportion of slow I fibers than NM muscles as early as late gestation [31]. Proteomics showed increased expression of fast proteins and lowered expression of slow proteins in the ST muscle of DM bull calves [20]. Accordingly, our study revealed gene expression profiles that may be molecular signatures of the DM fast-type phenotype. Such features are likely to originate from a high proportion in the secondary generation of muscle fibers following the loss of myostatin function, as early as Day 110 of fetal age in cattle [8].

#### ECM-specific genes

It is notable that genes encoding ECM/cell interaction proteins, e.g. *COL1*A1, *COL1A2 *and *COL3A1 *(*collagen type I *and *III)*, *FN1 (*murine and human *fibronectin 1)*, *LAMB1 (laminin beta 1)*, and *BGN *(*biglycan*, a known TGFβ target gene [32]) are down-regulated in DM muscles. ECM has a profound influence on the differentiation of muscle cells and can regulate growth factor function in skeletal muscle (for a review, see [33]). Our data are in accordance with the reduced connective tissue content in DM muscles [34,35] and the decreased expression in DM fetal muscles of the major collagen isoforms, namely the *type I *and *III collagens*, as shown by *in situ *experiments [36]. Conversely, a novel result of the present study was the up-regulation of genes encoding type IV collagen, the major structural component of basement membrane surrounding and supporting skeletal muscle cells, and PLOD3, an enzyme playing an essential role in the supramolecular assembly of collagen IV [37]. Collagen IV was shown to be located mainly in the endomysium whereas collagen I and III isoforms are located both in the perimysium and endomysium [36]. Thus, the ultrastructure of the DM muscle connective tissue could differ from that of NM at both the endomysium and the perimysium levels.

#### Gene marker of adipocyte differentiation

Lastly, the most novel and interesting result of our study is the down-regulated expression of *C1QTNF3 *(Table 4), the differential expression of which was confirmed by RT-PCR (Table 5). This gene encodes an adipocyte differentiation marker with striking homologies with adiponectin [38]. Similarly, decreased *C1QTNF3 *expression was detected in DM cows (our unpublished data). The finding that a loss-of-function mutation of *myostatin *is associated with decreased adipocyte differentiation is consistent with the low fat depots in *myostatin*-null mice [39] and in DM cattle [35]. Recently, myostatin has been shown to promote the commitment of mesenchymal stem cells to the adipogenic lineage [40]. Potts et al. [26] have also reported decreased expression of *HMGA2*, a transcription factor involved in fat cell proliferation, in DM cattle embryos. The expression of another adipocyte differentiation marker (*A-FABP*) was found to be lower in the muscles of DM Belgian Blue bulls compared to bulls with no *myostatin *mutation [41], illustrating a reduction in intramuscular adipocyte numbers. Since the number of intramuscular adipocytes is a major factor in determining muscle marbling of beef [42], it could partly explain the lower intramuscular fat development of DM muscles [35].

## Conclusion

In conclusion, transcriptomic analysis enabled us to demonstrate that the biological traits of DM muscles are associated with specific gene profiles at the time of fiber differentiation and/or specialization in late fetuses. On the one hand, our results confirm previous data obtained postnatally by classical biochemical and molecular biological approaches. On the other hand, they reveal altered gene expression in the three major muscle compartments, namely fibers, connective tissue and intramuscular adipose tissue. This may help us to understand how *myostatin *loss-of-function affects so many qualitative properties of muscles. Lastly, this study revealed novel putative myostatin targets, e.g. ECM constituents such as *type IV collagen, C1QTNF3 *mainly associated with adipose tissue development, and genes encoding transcription factors (ZFH1XB, ...). Work is in progress to determine whether these genes are direct or indirect targets targets of myostatin throught the examination of putative gene networks.

## Methods

### Animals

Animals were obtained as described previously [43]. Only 260-day-old fetuses were used in this study. Three DM fetuses were obtained by artificial insemination of Charolais heifers by transplantation of frozen embryos of strain INRA95. This strain comprised a mixture of breeds and the transplanted embryos contained around 75% Charolais. Three normal Charolais fetuses were obtained by artificial insemination of Charolais heifers using Charolais sperm from non-DM sires. After slaughter, the fetuses were collected and the *semitendinosus *muscle was excised, snap frozen and stored at -80°C prior to analysis.

### Microarray experiments

Transcriptomic analysis was performed with a microarray of around 6,000 genes expressed in muscle; these so called "Myochips" are available from West Genopole [44]. The Myochips were made from a set of relevant genes (probes). They were composed of 919 control spots and 6,473 oligonucleotides (50-mers) representing genes preferentially and/or differentially expressed in normal and diseased striated mice or human muscles and heart. These genes encode proteins belonging to all the main functional categories in striated muscle [44]. They were classified at the biological process level according to Gene Ontology annotations. Three replicates of each gene were spotted on to Myochips, which allowed detailed statistical studies of the reproducibility of the hybridization experiments. Microarray experiments were performed according to recently proposed standards (MIAME consortium [45]). Data were incorporated into the BASE database and the NCBI Gene Expression Omnibus (GEO) [46] and are accessible through GEO Series accession number GSE5456.

The protocol used was that described in the DNA Chips platform protocols. Total RNA was extracted from muscle tissue samples with TRIZOL^® ^reagent (Life Technologies) according to the manufacturer's recommendation. Each individual sample was compared to a reference pool consisting of skeletal muscle transcripts isolated from the *semitendinosus *muscles of five 260-day-old NM fetuses and of 16 Charolais heifers. Total RNA (15 μg) was reverse transcribed using the CyScribe cDNA Post Labelling kit (Amersham Pharmacia Biotech) using random nanomers for priming. During reverse transcription, aminoallyl-dUTP was incorporated to perform labeling with cyanins (Cy5 for the reference sample and Cy3 for individual samples). Four chips were hybridized per sample comparison. After washing, the chips were scanned on an Affymetrix 428™ Array Scanner.

### Data analysis

After acquisition, the scanned images were analyzed using GenePix Pro V6 software (Axon instrument, Inc). Raw signal intensity data were normalized using the MADSCAN *lowess fitness *method [47]. In order to identify differentially expressed genes, the Cy3/Cy5 ratios were statistically analyzed using SAM [48]. Data were also analyzed by standard analysis of variance (ANOVA) using GeneANOVA software [49]. In order to identify similar expression patterns, gene expression data were analyzed with Genesis [50] using hierarchical clustering (Average linkage and Euclidian distance).

Putatively involved pathways were explored using the FatiGoplus web tool [51], which is an extension of FatiGO [52] to other types of relevant biological knowledge, and using the Bibliosphere Pathway Edition web tool of the Genomatix Suite [53].

Differential expression was checked by RT-PCR for some genes, e.g. *TPM3 *(Fw primer: CTGGAGGAGGAGCTGAAGAA; Rv primer: CAGCTTGGCTACCGATCTCT), *SERCA2 *(Fw primer: TCTGCCTGTCGATGTCACTC; Rv primer: GTTGCGGGCCACAAACTT), *MYH7 *(Fw primer: CACCAACCTGTCCAAGTTCC; Rv primer: ACTGGGAGCTTCAGTTGCAC), PLN (Fw primer: ACTTGGCTGGCAGCTTTTTA; Rv primer: ACTGGGATTGCAGCAGAACT), *C1QTNF3 *(Fw primer: CGCTCACTTCACCAATCAGA; Rv primer: TGCATGGTTGCTGGATGTAT), *MSTN *(Fw primer: GTCTGCCCTTGTTAATTACCAG; Rv primer: CATCAGAGCAACTTGAGGTGG), *COL1A1 *(Fw primer: CACCTACCACTGCAAGAACAG, Rv primer: GAATGCACTTTTGGTTTTTGGTG), *BGN *(Fw primer: CACCTTGGTGATGTTGTTGG; Rv primer: TCTCGTCCGCTACTCCAAGT), *PLOD3 *(Fw primer: AACGGGGCTTTAGATGAGGT; Rv primer: CGTGGTACACCTCGTTGTTG) using a LightCycler FastStart DNA Master SYBR Green I kit (Roche Diagnostics GmBH, Mannheim, Germany) according to the following procedure: Mg^2+ ^added at a final concentration of 2 mM; pre-incubation step at 95°C for 10 min; amplification step (40 cycles) including denaturation at 95°C for 10 s, annealing at 60°C for 7 s, extension at 72°C for 10 s; melting curve including denaturation at 95°C for 0 s, annealing at 70°C for 20 s, continuous melting at 98°C for 0 s (slope = 0.1°C/s); cooling step at 40°C for 30 s. For *MSTN*, annealing was at 56°C. Results are expressed in pg/μmol relative to a standard curve of purified cDNA for each gene. Expression data from the 2 homozygote DM fetuses only and the 3 NM fetuses were analyzed using the Mann Whitney U Test and the difference was declared significant for U= 0 (p = 5%). For *MSTN*, annealing was at 56°C. Results are expressed as Ct values.

## List of abbreviations used

SAM: Statistical Analysis of Micro-arrays; ECM: Extra Cellular Matrix; DNA: Desoxyribo Nucleic Acid; NM: Normally-Muscled cattle; DM: Double-Muscled cattle, PCA: Principal Components Analysis; FC: Fold Change; FDR: False Discovery Rate.

## Authors' contributions

I. Cassar-Malek and J-F. Hocquette conceived the experimental design. I. Cassar-Malek and F. Passelaigue carried out the microarray experiments. F. Passelaigue and C. Bernard were actively involved in data analyses and interpretation. J. Léger  conceived and provided the arrays, as well as the informatic tools for array analyses. He also provided useful advice for data analysis and interpretation. I Cassar-Malek wrote the manuscript. All authors read the manuscript, significantly contributed either to the presentation, the interpretation or the discussion of the results, and were highly involved in writingthe redaction of the manuscript. They all read and approved the final manuscript. J-F Hocquette led the program entitled "Identification of genes involved in beef quality" with grants from the "Commissariat à l'Aménagement et au Développement Economique du Massif Central" (France) in which this study was included.

## Figures and Tables

**Table 1:** KEGG pathway results of the genes of which the expression varied according to whether one or two functional myostatin allele(s) were present.

**KEGG Entity**	**Genes list**	**Percentage of annoted genes list**
Focal adhesion	FN1, ITGA4, ITGA3, ARHA, COL1A1, CAPN3, CAV3, RRAS2	5.6
Axon guidance	ABLIM1, SEMA3D, ARHA, RRAS2, Limk1, NFAT5	4.2
Regulation of actin cytoskeleton	FN1, ITGA4, ITGA3, ARHA, RRAS, Limk1	4.2
Neuroactive ligand-receptor interaction	ADRA1B, GPR50, EDN2, EDNRA, THRA, GPR72	4.2
Calcium signaling pathway	ADRA1B, CAMK4, EDNRA, NFAT5, PRKACG	3.5
Cell cycle	Mcm6, HDAC4, SMC1L2, E2F1	2.8
Wnt signaling pathway	ARHA, CTBP1, NFAT5, PRKACG	2.8
Insulin signaling pathway	TSC, RRAS2, FASN, PRKACG	2.8
Glutathione metabolism	G6PD, Gclc, Gpx3, MGST1	2.8
ECM-receptor interaction	FN1, ITGA4, ITGA3, COL1A1	2.8
Cell adhesion molecules (CAMs)	ITGA4, NECL1, NCAM1	2.1
Ribosome	Rps29, RPL37A, RPL28	2.1
MAPK signaling pathway	DUSP9, RRAS2, PRKACG	2.1
T cell receptor signaling pathway	ARHA, RRAS2, NFAT5	2.1

Expression data were filtered for 20% missing values, processed by ANOVA and by Genesis. A total of 189 genes were selected from 8 clusters for similar regrouping according to the homozygote or heterozygote status of the Q204X mutation. They were submitted to FatiGo+ search. Out of the 189 genes examined, 142 had a GO annotation, 42 were unknown and 5 had.... more than one ensemble ID. Amongst the 51 KEGG pathways found, only the KEGG pathway results including several genes are presented.

**Table 2:** Numbers of genes declared by SAM to be differentially expressed (FC ≥ 1.4, FDR < 5‰) in the *Semitendinosus *muscle of DM *vs *NM fetuses.

	**Down-regulated genes**	**Up-regulated genes**
Data set 1 (total)	134	156
Data set 2 (reduced)	178	242
Genes in common	125	156

Dataset 1 comprised expression data from 3 fetuses per group; dataset 2 excluded the data from the DM108 heterozygote fetus and from the NM0423 fetus.

**Table 3:** Examples of up-regulated genes in DM *semitendinosus.*

**Gene symbol**	**Gene name**	**Fold change**	**Homology**
2610103N14Rik	Slc16a10 Solute carrier family 16	2.76	53%
2410044K02Rik	Thoc3 THO complex 3	2.64	96%
SURF1	Surfeit 1	2.54	88%
LOC58504	Hypothetical protein from clones 23549 and 23762	2.52	86%
Slc26a4	Solute carrier family 26, member 4	2.48	ND
FLJ13855	Hypothetical protein FLJ13855	2.36	94%
C8orf1	Chromosome 8 open reading frame 1	2.27	92%
MYBPH	Myosin binding protein H	2.27	90%
CXORF6	Chromosome × open reading frame 6	2.19	50%
ACCN2	Amiloride-sensitive cation channel 2, neuronal	2.17	ND
MYOM2	Myomesin (M-protein) 2, 165kDa	2.14	84%
Olfr51	Olfactory receptor 51	2.13	51%
Fancg	Fanconi anemia, complementation group G	2.12	80%
4631402N15Rik	Usp38 Ubiquitin specific protease 38	2.10	56%
Rusc2	RUN and SH3 domain containing 2	2.05	80%
C:3410	EST	2.02	ND
PDF	Peptide deformylase-like protein	2.01	86%
COL4A2	Collagen type, IV, alpha2	1.84	86%
PLOD3	Procollagen-lysine,2-oxoglutarate 5-dioxygenase 3	1.80	92%
FURIN	Furin	1.63	86%
ZFHX1B	Zinc finger homeobox 1b	1.58	100%

Genes were declared differentially expressed by SAM (FDR < 5‰) and by GeneANOVA with a p-value < 2% from dataset 2. Percentage homologies between spotted oligonucleotides and bovine sequences were determined using BLASTN and BLASTX searches. ND: None determined (unknown bovine sequence).

**Table 4:** Examples of down-regulated genes in DM *semitendinosus*.

**Gene**	**Gene name**	**Fold change**	**Homology**
Tncc	Troponin C, cardiac/slow skeletal	6.22	94%
PLN	Phospholamban	5.48	98%
ALPHA-1COLLAGEN-a	Alpha-1 collagen type 1	5.22	98%
MYH7-a	Myosin, heavy polypeptide 7, cardiac muscle, beta	4.86	96%
TNNC1	Troponin C, slow	4.71	94%
Tpm3	Tropomyosin 3	3.38	94%
MYH7-b	Myosin, heavy polypeptide 7, cardiac muscle, beta	3.37	90%
TNNC1	Troponin C, slow	3.32	92%
COL3A1	Collagen, type III, alpha 1	2.98	96%
ALPHA-1COLLAGEN-b	Alpha-1 collagen type 1	2.97	98%
VIM	Vimentin	2.76	94%
COL1A2	Collagen, type I, alpha 2	2.64	84%
GPR72	G protein-coupled receptor 83	2.52	ND
S100A4	S100 calcium binding protein A4	2.38	92%
COL1A1	Collagen, type I, alpha 1	2.26	80%
GL004	Chromosome 2 open reading frame 33	2.25	94%
Fn1	Fibronectin 1	2.11	90%
MYL2	Myosin, light polypeptide 2, regulatory, cardiac, slow	2.08	92%
MRPL15	Mitochondrial ribosomal protein L15	2.01	84%
hypothLOC150928	Hypothetical LOC150928	2.01	98%
SERCA2	ATPase, CA++ transporting, cardiac muscle	2.00	94%
MB	Myoglobin	1.90	90%

Genes were declared differentially expressed by SAM (FDR < 5‰) and by GeneANOVA with a p-value < 1‰ from dataset 2. Percentage homologies between spotted oligonucleotides and bovine sequences were determined using BLASTN and BLASTX searches. ND: none determined (unknown bovine sequence). -a, -b indicate two different oligonucleotides designed for a same gene.

**Table 5:** Validation of differential expression by real time PCR.

**Gene**	**Ratio NM/DM by array analysis**	**Ratio NM/DM by RT-PCR**
TPM3	3.38*	4.45*
ATP2A2	2*	2.85*
MYH7	4.86-*3.37*	3.28*
C1QTNF3	1.87*	2.97*
PLN	5.48*	4.31*
COL1A	5.22*-2.98*	10
BGN	1.79*	5.05*
PLOD3	1.80*	1.4*

Results are means of duplicate experiments. *All differential expressions are statistically significant at P < 5% for array experiments (using SAM analysis; comparison of 2 NM vs 2DM animals) or by real-time RT-PCR (using the Mann Whitney U Test).

**Table 6:** Main KEGG pathways involving the genes of which expression was either down- or up-regulated in DM *semitendinosus*.

**KEGG Entity**	**Down-regulated genes list**	**% of list**	**Up-regulated genes list**	**% of list**
Ribosome	RPS3A, RPS15A, Rpl30, RPS20, UBA52, RPL32, Rps24	14.29	No genes	0
Oxidative phosphorylation	ATP5A1, ATP5E, COX5A, Cox5b, ATP5F1, ATP5G2, NDUFS1	14.29	No genes	0
Insulin signaling pathway	No genes	0	MRAS, PRKAA1, TSC, RRAS2, PRKACG	10.2
ECM-receptor interaction	Fn1, COL1A1, COL1A2, Sdc4, LAMB1, COL3A1	12.24	ITGA3	2.0
ATP synthesis	ATP5A1, ATP5E, ATP5F1, ATP5G2	8.16	No genes	0
Cytokine-cytokine receptor interaction	No genes	0	TNFRSF14, LTB, CCL18, IL21R	8.2
Wnt signaling pathway	WNT6	2.04	Porcn, WNT8B, CTBP1, PRKACG	8.2
Axon guidance	No genes	0	MRAS, RRAS2, Limk1	6.2
Calcium signaling pathway	ATP2A2, PLN, Cacna1i, GNAS, Erbb4	10.2	CACNA1F, PRKACG	4.1

Genes were declared differentially expressed by SAM (FDR < 5‰) and by GeneANOVA with a p-value < 1‰ from the reduced dataset. The gene lists were submitted to FatiGO+ analysis and compared for KEGG pathway.
